# Enhanced Enforcement of Laws to Reduce Alcohol Overservice Among Licensed Establishments in New Mexico, 2004–2008

**DOI:** 10.5888/pcd15.180067

**Published:** 2018-12-06

**Authors:** Laura E. Tomedi, Jim Roeber, Ziming Xuan, Dafna Kanny, Robert D. Brewer, Timothy S. Naimi

**Affiliations:** 1 Epidemiology and Response Division, New Mexico Department of Health, Santa Fe, New Mexico; 2 Department of Community Health Sciences, Boston University School of Public Health, Boston Massachusetts; 3 National Center for Chronic Disease Prevention and Health Promotion, Centers for Disease Control and Prevention, Atlanta, Georgia; 4 Section of General Internal Medicine, Boston Medical Center, Boston Massachusetts

## Abstract

Limited information exists about the effectiveness of interventions to enforce laws prohibiting alcohol sales to intoxicated patrons in licensed establishments. New Mexico Behavioral Risk Factor Surveillance System data were used to evaluate an intervention on binge drinking intensity in licensed (eg, bars) versus unlicensed (eg, homes) locations. The proportion of binge drinkers in licensed locations who consumed 8 or more drinks on a binge drinking occasion decreased from 42.1% in 2004–2005 to 22.6% in 2007–2008 (adjusted odds ratio, 0.4; 95% confidence interval, 0.2–0.9), while the proportion in unlicensed locations was essentially unchanged. Enhanced enforcement of overservice laws may reduce excessive drinking in licensed establishments.

## Objective

Binge drinking accounts for a large proportion of alcohol consumption and increases the risk of chronic diseases (1–3). Most states prohibit the sale of alcohol to intoxicated patrons (overservice) (4), but these laws are poorly enforced. Beginning in 2006, New Mexico increased overservice enforcement in licensed locations (eg, bars) throughout the state, with citations increasing from 55 during January through June 2006 to 203 during January through June 2007. The objective of this study was to evaluate the effect of this enhanced enforcement on binge drinking intensity among adults drinking in licensed on-premise locations (eg, bars) compared with adults drinking in unlicensed locations (eg, homes).

## Methods

We conducted a multiple time period, cross-sectional study using alcohol consumption data from the New Mexico Behavioral Risk Factor Surveillance System (NMBRFSS), a random-digit–dialed telephone survey of adults on health-related risk behaviors (5). Because the intervention occurred in 2006, data from 2004–2005 and 2007–2008 were used to compare the pre-intervention period to the during/post-intervention period, respectively. In 2004–2005, binge drinking was defined as 5 or more drinks on at least 1 occasion in the past 30 days. In 2007–2008, binge drinking was defined as 5 or more drinks for men or 4 or more drinks for women on at least 1 occasion in the past 30 days (6).

We calculated binge intensity from the number of drinks binge drinkers consumed by type of alcoholic beverage (eg, beer, wine, liquor) during their most recent binge episode. Adults with missing information or who reported drinking fewer than 5 drinks during their most recent binge episode were excluded.

Respondents were asked about the location of their last binge episode (ie, at home, at another person’s home, at a restaurant or banquet hall, at a bar or club, or at a public place). For this analysis, these drinking locations were then dichotomized into licensed locations (restaurants, bars, or clubs) and unlicensed locations (other locations).

The proportion of binge drinkers who consumed 8 or more drinks (the average binge drinking intensity in the United States [7] and in New Mexico in 2004–2005) was calculated by drinking location. The odds ratio of consuming 8 or more drinks on a binge drinking occasion by time period was assessed by logistic regression and adjusted for race/ethnicity (white, non-white, Hispanic), age (18–34, ≥35), annual household income (<$25,000, ≥$25,000), educational status (high school diploma or less, more than high school diploma), and marital status (married, not married). The analyses were stratified by sex and age. The analysis adjusted for complex survey design by using Stata/MP 14.0 (StataCorp LLC). This study is public health practice and did not require institutional review board review (www.cste2.org/webpdfs/CSTEPHResRptHodgeFinal.5.24.04.pdf).

## Results

A total of 995 binge drinkers responded to the questions on beverage-specific alcohol consumption and drinking location in 2004–2005, and 1,008 binge drinkers responded to these questions in 2007–2008. After exclusions, 929 respondents in 2004–2005 and 757 respondents in 2007–2008 were included in the analysis. The exclusions were primarily women who reported consuming 4 drinks.

Between 2004–2005 and 2007–2008, the average number of binge drinks consumed by adult binge drinkers in licensed locations decreased from 8.2 drinks (95% confidence interval [CI], 7.4–8.9) to 7.2 drinks (95% CI, 6.3–8.0), whereas the average number of drinks consumed by binge drinkers in unlicensed locations remained stable (8.0 drinks [95% CI, 7.6–8.5] in 2004–2005 and 7.8 drinks [95% CI, 7.3–8.3] in 2007–2008). Similarly, the percentage of binge drinkers who consumed 8 or more drinks in licensed locations during their last binge drinking episode decreased from 42.1% in 2004–2005 to 22.6% in 2007–2008, a 46.3% relative decline (AOR of consuming ≥8 drinks, 0.4; 95% CI, 0.2–0.9) (Table). In contrast, the proportion of adult binge drinkers who consumed 8 or more drinks in unlicensed locations was unchanged between these time periods (37.8% versus 37.9%, AOR, 1.1; 95% CI, 0.8–1.5).

**Table:** Percentage^a^ of Binge Drinkers Who Consumed ≥8 Drinks, by Location^b^, in Most Recent Binge Drinking Episode, for Total Sample and by Sex and Age, New Mexico, Behavioral Risk Factor Surveillance System, 2004–2005 and 2007–2008

Category	2004–2005		2007–2008		% Change	AOR^d^ (95% CI)
	No.^c^	≥8 Drinks, % (95% CI)	No.^c^	≥8 Drinks, % (95% CI)		
**Total Sample**						
Licensed locations	195	42.1 (33.5–51.3)	121	22.6 (14.9–32.7)	−46.3	0.4 (0.2–0.9)
Unlicensed locations	734	37.8 (33.2–42.6)	636	37.9 (32.6–43.5)	0.3	1.1 (0.8–1.5)
**Sex**						
**Women**						
Licensed locations	63	26.2 (15.2–41.3)	50	17.5 (7.6–35.3)	−33.2	0.7 (0.2–2.0)
Unlicensed locations	202	32.5 (23.8–42.6)	155	33.6 (24.3–44.3)	3.4	1.3 (0.7–2.5)
**Men**						
Licensed locations	132	47.6 (37.0–58.4)	71	25.5 (15.6–38.7)	−46.4	0.4 (0.1–0.8)
Unlicensed locations	532	39.4 (34.1–44.9)	481	38.9 (32.9–45.3)	−1.3	1.1 (0.8–1.6)
**Age^e^**						
**18–34 y**						
Licensed locations	95	55.5 (43.0–67.3)	52	25.5 (14.4–41.1)	−54.1	0.3 (0.1–0.7)
Unlicensed locations	284	47.5 (40.2–54.8)	200	47.2 (38.3–56.3)	−0.6	1.1 (0.7–1.8)
**≥35 y**						
Licensed locations	100	23.8 (15.2–35.2)	69	18.2 (10.2–30.3)	−23.5	0.5 (0.2–1.4)
Unlicensed locations	448	26.6 (22.0–31.8)	435	28.41 (23.1–34.4)	6.8	1.2 (0.8–1.8)

Abbreviations: AOR, adjusted odds ratio; CI, confidence interval.

a Weighted percentage and CI of binge drinkers who reported consuming ≥8 drinks on their most recent binge drinking episode.

b Licensed location = bar, club, restaurant; unlicensed location = home, another’s home, public place.

c Unweighted number of respondents who reported binge drinking in each category.

d AOR of consuming ≥8 drinks among binge drinkers in 2007–2008 compared with 2004–2005. Analyses were adjusted for sex, race/ethnicity (white, non-white, Hispanic), age (18–34, ≥35), annual household income (<$25,000, ≥$25,000), educational status (high school diploma or less, more than high school diploma), and marital status (married, not married), except when these variables were included in stratified analyses.

e Two respondents were missing values for age.

The percentage of male binge drinkers in licensed locations who consumed 8 or more drinks on a binge drinking occasion decreased from 47.6% in 2004–2005 to 25.5% in 2007–2008 (AOR, 0.4; 95% CI, 0.1–0.8), whereas the percentage of male binge drinkers in unlicensed locations who consumed 8 or more drinks changed little (39.4% vs 38.9%, respectively; AOR, 1.1; 95% CI, 0.8–1.6). The percentage of binge drinkers aged 18 to 34 years in licensed locations who consumed 8 or more drinks also decreased significantly from 55.5% in 2004–2005 to 25.5% in 2007–2008 (AOR, 0.3; 95% CI, 0.1–0.7).

## Discussion

This study found that enhanced enforcement of a law prohibiting overservice was associated with a nearly 50% reduction in the proportion of binge drinkers who had 8 or more drinks in licensed locations during their most recent binge drinking episode. This resulted in an average reduction of 1 drink at last binge episode. Significant reductions in binge drinking intensity were specifically found among men and among people aged 18 to 34 years, groups that are known to be at greater risk of binge drinking at high intensity (8). These declines occurred in the absence of similar declines in binge drinking intensity among those drinking in private homes or other unlicensed locations, suggesting that the observed decline in binge drinking intensity in licensed drinking locations was associated with the enhanced enforcement.

This study has several limitations. First, it is based on a repeat cross-sectional survey, which precluded the assessment of changes in binge drinking intensity among individual binge drinkers. Second, surveys are subject to nonresponse bias and inaccurate reporting of alcohol consumption; however, response rates for the NMBRFSS were similar during the preintervention and postintervention periods. Third and finally, these results may not be generalizable outside New Mexico. Despite these limitations, the findings of this study indicate that enhanced enforcement of overservice laws may reduce binge drinking intensity among adults drinking in licensed locations.

## References

[1] World Health Organization. Global status report on alcohol and health — 2014. Geneva (CH): World Health Organization; 2014.

[2] Shield KD , Parry C , Rehm J . Chronic diseases and conditions related to alcohol use. Alcohol Res 2013;35(2):155–73. 24881324 10.35946/arcr.v35.2.06 PMC3908707

[3] Rehm J , Room R , Graham K , Monteiro M , Gmel G , Sempos CT . The relationship of average volume of alcohol consumption and patterns of drinking to burden of disease: an overview. Addiction 2003;98(9):1209–28. 10.1046/j.1360-0443.2003.00467.x 12930209

[4] Rammohan V , Hahn RA , Elder R , Brewer R , Fielding J , Naimi TS , Effects of dram shop liability and enhanced overservice law enforcement initiatives on excessive alcohol consumption and related harms: two Community Guide systematic reviews. Am J Prev Med 2011;41(3):334–43. 10.1016/j.amepre.2011.06.027 21855749

[5] Centers for Disease Control and Prevention. Behaviorial Risk Factor Surveillance System. http://www.cdc.gov/brfss/. Accessed August 8, 2016.

[6] Centers for Disease Control and Prevention. Fact sheets — binge drinking. http://www.cdc.gov/alcohol/fact-sheets/binge-drinking.htm. Accessed August 15, 2016.

[7] Naimi TS , Nelson DE , Brewer RD . The intensity of binge alcohol consumption among U.S. adults. Am J Prev Med 2010;38(2):201–7. 10.1016/j.amepre.2009.09.039 20117577

[8] Kanny D , Liu Y , Brewer RD , Lu H ; Centers for Disease Control and Prevention (CDC). Binge drinking — United States, 2011. MMWR Suppl 2013;62(3):77–80. 24264494

